# Factors Associated With Psychological Distress in a Community-Dwelling Japanese Population: The Ohsaki Cohort 2006 Study

**DOI:** 10.2188/jea.JE20080076

**Published:** 2009-11-05

**Authors:** Shinichi Kuriyama, Naoki Nakaya, Kaori Ohmori-Matsuda, Taichi Shimazu, Nobutaka Kikuchi, Masako Kakizaki, Toshimasa Sone, Fumi Sato, Masato Nagai, Yumi Sugawara, Munira Akhter, Mizuka Higashiguchi, Naru Fukuchi, Hideko Takahashi, Atsushi Hozawa, Ichiro Tsuji

**Affiliations:** 1 Division of Epidemiology, Department of Public Health and Forensic Medicine, Tohoku University Graduate School of Medicine, Sendai, Japan; 2 Epidemiology and Prevention Division, Research Center for Cancer Prevention and Screening, National Cancer Center, Tokyo, Japan

**Keywords:** cross-sectional, K6, population-based, psychological distress

## Abstract

**Background:**

In Asia, there has been no population-based epidemiological study using the K6, a 6-item instrument that assesses nonspecific psychological distress.

**Methods:**

Using cross-sectional data from 2006, we studied 43 716 (20 168 men and 23 548 women) community-dwelling people aged 40 years or older living in Japan. We examined the association between psychological distress and demographic, medical, lifestyle, and social factors by using the K6, with psychological distress defined as 13 or more points out of a total of 24 points.

**Results:**

The following variables were significantly associated with psychological distress among the population: female sex, young and old age, a history of serious disease (hypertension, diabetes mellitus, stroke, myocardial infarction, or cancer), current smoking, former alcohol drinking, low body mass index, shorter daily walking time, lack of social support (4 of 5 components), and lack of participation in community activities (4 of 5 components). Among men aged 40 to 64 years, only “lack of social support for consultation when in trouble” and a history of diabetes mellitus remained significant on multivariate analysis. Among men aged 65 years or older, age was not significantly associated with psychological distress, and the significant association with current smoking disappeared on multivariate analysis. Among women aged 40 to 64 years, a history of stroke was not associated with psychological distress. Among women aged 65 years or older, the significant association with current smoking disappeared on multivariate analysis.

**Conclusions:**

A number of factors were significantly associated with psychological distress, as assessed by the K6. These factors differed between men and women, and also between middle-aged and elderly people.

## INTRODUCTION

Mental health is an important component of overall well-being. About 14% of the global disease burden has been attributed to mental illness, mostly due to the chronically disabling nature of depression and other common mental disorders.^1^^,^^2^ Although numerous studies have produced systematic evidence regarding the risk factors for physical health, the understanding of factors related to mental health, particularly in Asian countries, is still limited.^2^

In 2002, in an attempt to devise a method to easily assess mental health in general population surveys, Kessler and colleagues developed a scale of nonspecific psychological distress—the K6—that comprises only 6 questions.^3^ The K6 was originally developed to identify persons with a high likelihood of developing mental conditions, such as depression and mood or anxiety disorders.^4^ However, the K6 and the K10 (the K6 plus 4 additional questions related to symptoms of distress) have also been used to estimate the prevalence of nonspecific psychological distress in general population surveys,^5^ and as part of the World Health Organization’s World Mental Health Surveys.^6^ Although it is brief enough to be added to lengthy general health questionnaires, a major limitation of the K6 is that it does not provide information on the particular psychiatric diagnosis or diagnoses a respondent may have. Nevertheless, researchers have begun to use the K6 for studies in clinical settings,^7^ as well as in epidemiological studies^8^^,^^9^ and large, nationally representative surveys. Despite the frequent use of the K6, no population-based epidemiological study has used this scale to clarify the factors associated with mental health in Asian countries.

The objective of the present study was to use the K6 to identify factors associated with psychological distress in a community-dwelling Japanese population. We also briefly describe the overall design of the study, as this is the first report from a new prospective cohort study, the Ohsaki Cohort 2006 Study.

## METHODS

### Study design, setting, and participants

The Ohsaki Cohort 2006 Study is a prospective cohort study, from which we analyzed cross-sectional data from a baseline survey. The source population for the baseline survey comprised community-dwelling individuals aged 40 years or older who were included in the Residential Registry for Ohsaki City, Miyagi Prefecture, northeastern Japan, as of December 1, 2006. The Residential Registry identified 78 101 persons (36 397 men; 41 704 women) living in the area.

The baseline survey was conducted from December 1 to December 15, 2006. A questionnaire was distributed by the heads of individual administrative districts to individual households, and returned by mail. Of the 78 101 persons, 866 were ineligible due to death, move-out, or hospitalization, yielding an eligible population of 77 235. The baseline questionnaires (described below) were collected from 50 210 persons, and valid responses were received from 49 855 (22 547 men and 27 308 women), who ultimately formed the study population of cohort participants. Among the study population, 26 512 persons (53.2%) were aged 40 to 64 years, and 23 343 (46.8%) were aged 65 years or older. The response rate was calculated by dividing the study population by the total eligible population, yielding 64.5%. The corresponding response rates, with respect to sex and age categories, were 54.9% and 60.4% among men and women aged 40 to 64 years, respectively, and 77.1% and 73.2% among men and women aged 65 years or older, respectively.

When analyzing the prevalence of psychological distress and its associations with demographic, medical, lifestyle, and social factors, we excluded participants for whom K6 data were missing (*n* = 6139). Consequently, the analyzed population comprised 43 716 participants (20 168 men and 23 548 women; 56.6% of the eligible population).

### Baseline survey

The baseline questionnaires for persons aged 40 to 64 years consisted of the following details in sequence: (1) history of diseases, (2) family history of diseases, (3) health status during the preceding year, (4) smoking status, (5) alcohol drinking status, (6) dietary habits,^10^ (7) job status and educational status, (8) present and past body weight and height, (9) health status in general, (10) sports and exercise,^11^^,^^12^ (11) psychological distress (K6),^3^^,^^4^ (12) social support,^13^ (13) participation in community activities, (14) dental status, and (15) reproductive factors (among women).

The baseline questionnaires for persons aged 65 years or older consisted of the following details in sequence: (1) a frailty checklist (the Kihon checklist),^14^ (2) history of diseases, (3) health status during the preceding year, (4) smoking status, (5) alcohol drinking status, (6) dietary habits,^10^ (7) past body weight and height, (8) health status in general, (9) pain, (10) daily activities, (11) sports and exercise,^11^^,^^12^ (12) psychological distress (K6),^3^^,^^4^ (13) social support,^13^ (14) participation in community activities, and (15) dental status.

Questionnaire items for persons aged 65 years or older were identical to those for persons aged 40 to 64 years, except that the former excluded family history of diseases, job status and educational status, present and past body weight and height, and reproductive factors in women, and included the frailty checklist, past body weight and height, pain, and daily activities.

### Measurement of psychological distress

The K6 was used as an indicator of psychological distress.^3^^,^^4^ The 6 questions were as follows: “Over the last month, how often did you feel: (1) nervous, (2) hopeless, (3) restless or fidgety, (4) so sad that nothing could cheer you up, (5) that everything was an effort, (6) worthless?” Participants were asked to respond by choosing “all of the time” (4 points), “most of the time” (3 points), “some of the time” (2 points), “a little of the time” (1 point), and “none of the time” (0 points). Total point score therefore ranged from 0 to 24. The K6 has been developed using modern psychometric theory and has been shown to be superior to some existing scales in brevity and psychometric properties.^3^^,^^4^^,^^15^ The Japanese version of the K6 has been recently developed, using the standard back-translation method, and has been validated.^16^ As suggested by Kessler and colleagues,^15^ we classified participants with scores of 13 points or more as having psychological distress.

### Measurement of other variables

The degree of social support available to each person was assessed by asking the following questions^13^: (1) Do you have someone with whom you can consult when you are in trouble?, (2) Do you have someone with whom you can consult when your physical condition is bad?, (3) Do you have someone who can help you with your daily housework?, (4) Do you have someone who can take you to a hospital when you do not feel well?, and (5) Do you have someone who can take care of you when you are ill in bed? This social support questionnaire consisted of 5 questions, each requiring an answer of yes or no. This questionnaire was only available in Japanese, and its validity and reliability were not evaluated.

The frailty checklist is a tool developed by the Japanese Ministry of Health, Labour, and Welfare to screen for frail persons and is designed to measure actual task performance.^14^ Researchers have also begun to use this tool in epidemiological surveys.^14^

### Ethical issues

The return of questionnaires completed by the participants was regarded as consent to participate in the study, which involved cross-sectional analysis of the baseline survey data and the longitudinal study of subsequent mortality and immigration. The study protocol was reviewed and approved by the Ethics Committee of Tohoku University Graduate School of Medicine.

### Statistical analysis

We used univariate and multivariate logistic regression analysis to calculate the odds ratios (ORs) for psychological distress (a K6 total score of ≥13 points) relative to demographic, medical, lifestyle, and social factors. In these analyses, we investigated the following factors: sex, age (40–44, 45–49, 50–54, 55–59, 60–64, 65–69, 70–74, 75–79, 80–84, ≥85 years), history of hypertension (yes, no), history of diabetes mellitus (yes, no), history of stroke (yes, no), history of myocardial infarction (yes, no), history of cancer (yes, no), smoking status (never, former, current), alcohol drinking (never, former, current), body mass index (kg/m^2^) calculated with self-reported weight and height; <18.5, 18.5–24.9, ≥25.0), daily walking time (<30 min/day, 30 min–1 hour/day, ≥1 hour/day), social support (yes, none), participation in community activities (yes, none). In the multivariate models, the above variables were all adjusted for each other. Analyses were repeated by stratifying the population by sex and age categories (40–64 years, 65 years or older). When analyzing the data for men and women aged 40 to 64 years, we further added current employment status (yes, no) and duration of education (≤12 years, >12 years) as covariates. All statistical analyses were performed with SAS version 9.1 (SAS Inc., Cary, NC, USA), and all statistical tests were 2-sided. A *P* value less than 0.05 was considered to indicate statistical significance.

## RESULTS

### Prevalence proportion, and univariate and multivariate analysis of psychological distress among the total population

The crude prevalence proportion of psychological distress in the analyzed population was 6.7% (2921/43 716; 95% confidence interval [CI], 6.5 to 6.9). Univariate analysis showed that the following were significantly associated with a higher prevalence of psychological distress: female sex, young and old age, a history of serious disease, a current smoking habit, a former alcohol drinking habit, low BMI, shorter daily walking time, lack of social support, and lack of participation in community activities.

After mutual adjustment for the variables shown in Table 1, women had approximately 1.6 times the odds of psychological distress, relative to men. There was a U-shaped association between age category (5-year categories from 40–44 to ≥85 years) and the prevalence of psychological distress, with a nadir for those aged 65 to 69 years.

**Table 1.** Univariate and multivariate analysis of the associations between psychological distress and demographic, medical, lifestyle, and social factors among the total study population^a^

Variables	No. of persons with psychological distress /No. of participants	Univariate OR (95% CI)	Multivariate OR (95% CI)^b^
Sex			
​ Male	1146/20 168	1.00 (referent)	1.00 (referent)
​ Female	1775/23 548	1.35 (1.25–1.46)	1.58 (1.41–1.76)

Age group (years)			
​ 40–44	316/3702	1.00 (referent)	1.00 (referent)
​ 45–49	380/4739	0.93 (0.80–1.09)	0.93 (0.79–1.09)
​ 50–54	390/5712	0.79 (0.67–0.92)	0.79 (0.67–0.93)
​ 55–59	398/6734	0.67 (0.58–0.79)	0.65 (0.56–0.77)
​ 60–64	226/4461	0.57 (0.48–0.68)	0.54 (0.45–0.65)
​ 65–69	240/5091	0.53 (0.45–0.63)	0.52 (0.43–0.63)
​ 70–74	296/5242	0.64 (0.54–0.76)	0.57 (0.47–0.68)
​ 75–79	281/4167	0.78 (0.66–0.92)	0.60 (0.50–0.72)
​ 80–84	214/2347	1.08 (0.90–1.29)	0.74 (0.60–0.91)
​ ≥85	180/1521	1.44 (1.19–1.75)	0.87 (0.69–1.08)

History of diseases			
​ Hypertension	907/12 658	1.11 (1.03–1.21)	1.17 (1.07–1.28)
​ Diabetes mellitus	319/3819	1.31 (1.16–1.48)	1.26 (1.11–1.44)
​ Stroke	156/1012	2.63 (2.21–3.14)	2.12 (1.76–2.57)
​ Myocardial infarction	122/1147	1.69 (1.40–2.05)	1.51 (1.23–1.86)
​ Cancer	225/2432	1.46 (1.27–1.68)	1.48 (1.28–1.73)

Smoking status			
​ Never	1443/22 219	1.00 (referent)	1.00 (referent)
​ Former	553/9030	0.94 (0.85–1.04)	1.15 (1.01–1.31)
​ Current	701/9699	1.12 (1.02–1.23)	1.32 (1.17–1.49)

Alcohol drinking status			
​ Never	1187/17 041	1.00 (referent)	1.00 (referent)
​ Former	407/3633	1.69 (1.50–1.90)	1.49 (1.31–1.70)
​ Current	1156/20 840	0.78 (0.72–0.85)	0.94 (0.84–1.04)

Body-mass index			
​ <18.5 kg/m^2^	226/1803	2.12 (1.82–2.45)	1.59 (1.36–1.86)
​ 18.5–24.9 kg/m^2^	1689/26 610	1.00 (referent)	1.00 (referent)
​ ≥25.0 kg/m^2^	752/12 231	0.97 (0.89–1.06)	0.96 (0.87–1.05)

Time spent walking per day			
​ <30 min	1426/16 476	1.64 (1.49–1.80)	1.26 (1.14–1.40)
​ 30 min–1 hr	710/14 190	0.91 (0.82–1.02)	0.89 (0.79–0.99)
​ ≥1 hr	658/12 024	1.00 (referent)	1.00 (referent)

Lack of social support:			
​ (i) to consult when you are in trouble	873/5354	3.46 (3.18–3.77)	2.24 (1.97–2.56)
​ (ii) to consult when you are in bad physical condition	698/4167	3.39 (3.09–3.72)	1.24 (1.08–1.44)
​ (iii) to help with your daily housework	852/6701	2.47 (2.27–2.69)	1.12 (0.99–1.27)
​ (iv) to take you to a hospital	579/3834	2.86 (2.60–3.16)	1.27 (1.10–1.46)
​ (v) to take care of you	769/5563	2.71 (2.48–2.96)	1.42 (1.25–1.61)

No participation in community activities			
​ (i) Activities of neighborhood association	1952/22 109	2.26 (2.08–2.46)	1.27 (1.15–1.41)
​ (ii) Sports or exercise	2090/23 258	2.70 (2.47–2.95)	1.63 (1.47–1.81)
​ (iii) Volunteering	2307/28 871	2.48 (2.23–2.75)	1.17 (1.03–1.32)
​ (iv) Social gatherings	2016/22 568	2.48 (2.27–2.71)	1.31 (1.18–1.46)

Abbreviations: OR, odds ratio; CI, confidence interval.

^a^The K6 was used as an indicator of psychological distress,^3^^,^^4^ with a cut-off point of ≥13 out of 24 points.^15^

^b^In the multivariate models, all variables shown in Table 1 were adjusted for each other.

History of hypertension, diabetes mellitus, stroke, myocardial infarction, or cancer were all associated with a significantly higher prevalence of psychological distress in the multivariate models (Table 1). Among these diseases, a history of stroke was most strongly associated with psychological distress, and had more than 2 times the odds of psychological distress, relative to those who had no history of stroke.

A current smoking habit (vs never smoker), former smoking habit (vs never smoker), former alcohol drinking habit (vs never drinker), low BMI (vs normal BMI), and less daily walking time (vs time spent walking ≥1 hr) were associated with a higher odds for psychological distress, even in multivariate analysis (Table 1). In contrast, a moderate daily walking time (vs time spent walking ≥1 hr) was associated with a significantly lower odds.

Among the variables studied, lack of social support for consultation when in trouble was most strongly associated with a high prevalence of psychological distress in the multivariate models, although the association between other components of lack of social support and psychological distress was substantially attenuated in multivariate analysis (Table 1). The multivariate-adjusted OR (95% CI) for psychological distress associated with lack of social support for consultation when in trouble was 2.24 (1.97 to 2.56). The association of lack of participation in community activities with psychological distress was also attenuated, but lack of participation in neighborhood association activities, sports or exercise, volunteering, and community social gatherings were all associated with a higher prevalence of psychological distress, even in multivariate analysis.

### Stratified analysis by sex and age categories (40 to 64 years, 65 years or older)

Stratified analysis by sex and age categories (40 to 64 years, 65 years or older) yielded results similar to those for the participants as a whole (Table 1), but the statistically significant associations that had been observed between several factors and psychological distress disappeared in each stratum.

The statistically significant association between history of hypertension and psychological distress disappeared in all strata. Among men aged 40 to 64 years, there was loss of significant associations with a history of myocardial infarction, history of cancer, being a former smoker, spending less than 30 min per day walking, lacking social support for consultation when in a bad physical condition, lacking social support for transport to a hospital, lacking social support for receiving care, lack of participation in community activities in a neighborhood association, and lack of participation in community volunteer activities (Table 2). Among women aged 40 to 64 years, there was loss of the significant associations with a history of diabetes mellitus, history of stroke, spending less than 30 min per day walking, and lack of participation in community volunteer activities (Table 3).

**Table 2.** Multivariate analysis of the association between psychological distress and demographic, medical, lifestyle, and social factors among men aged 40 to 64 years^a^

Variables	No. of persons with psychological distress /No. of participants	Multivariate OR (95% CI)^b^
Age group (years)		
​ 40–44	128/1802	1.00 (referent)
​ 45–49	169/2299	0.99 (0.77–1.27)
​ 50–54	173/2781	0.85 (0.67–1.09)
​ 55–59	168/3269	0.65 (0.50–0.83)
​ 60–64	98/2108	0.55 (0.41–0.73)

History of diseases		
​ Hypertension	164/2529	1.17 (0.96–1.42)
​ Diabetes mellitus	98/1030	1.65 (1.30–2.10)
​ Stroke	24/170	2.42 (1.51–3.89)
​ Myocardial infarction	18/173	1.62 (0.94–2.76)
​ Cancer	23/307	1.26 (0.80–1.99)

Smoking status		
​ Never	97/2099	1.00 (referent)
​ Former	218/3940	1.19 (0.92–1.54)
​ Current	405/6087	1.38 (1.09–1.75)

Alcohol drinking status		
​ Never	107/1622	1.00 (referent)
​ Former	89/775	1.52 (1.11–2.07)
​ Current	531/9746	0.89 (0.71–1.11)

Body-mass index		
​ <18.5 kg/m^2^	39/266	2.20 (1.51–3.21)
​ 18.5–24.9 kg/m^2^	457/7749	1.00 (referent)
​ ≥25.0 kg/m^2^	235/4135	0.94 (0.79–1.12)

Time spent walking per day		
​ <30 min	330/4418	1.13 (0.94–1.37)
​ 30 min–1 hr	177/3807	0.80 (0.64–0.98)
​ ≥1 hr	217/3847	1.00 (referent)

Lack of social support:		
​ (i) to consult when you are ​ in trouble	339/2269	2.87 (2.30–3.58)
​ (ii) to consult when you are ​ in bad physical condition	258/1777	1.11 (0.87–1.41)
​ (iii) to help with your daily ​ housework	274/2205	1.23 (0.98–1.53)
​ (iv) to take you to a hospital	185/1340	1.14 (0.86–1.50)
​ (v) to take care of you	176/1261	1.28 (0.97–1.69)

No participation in community activities		
​ (i) Activities of neighborhood ​ association	425/5549	1.00 (0.83–1.21)
​ (ii) Sports or exercise	480/6078	1.35 (1.12–1.63)
​ (iii) Volunteering	545/7508	1.19 (0.95–1.48)
​ (iv) Social gatherings	476/5964	1.23 (1.02–1.50)

Abbreviations: OR, odds ratio; CI, confidence interval.

^a^The K6 was used as an indicator of psychological distress,^3^^,^^4^ with a cut-off point of ≥13 out of 24 points.^15^

^b^In the multivariate models, all variables shown in Table 2 were adjusted for each other.

**Table 3.** Multivariate analysis of the association between psychological distress and demographic, medical, lifestyle, and social factors among women aged 40 to 64 years^a^

Variables	No. of persons with psychological distress /No. of participants	Multivariate OR (95% CI)^b^
Age group (years)		
​ 40–44	188/1900	1.00 (referent)
​ 45–49	211/2440	0.87 (0.70–1.08)
​ 50–54	217/2931	0.75 (0.60–0.93)
​ 55–59	230/3465	0.65 (0.52–0.81)
​ 60–64	128/2353	0.51 (0.39–0.66)

History of diseases		
​ Hypertension	162/2225	1.10 (0.90–1.33)
​ Diabetes mellitus	49/567	1.19 (0.86–1.64)
​ Stroke	9/61	1.84 (0.87–3.91)
​ Myocardial infarction	8/48	3.00 (1.34–6.73)
​ Cancer	55/564	1.58 (1.17–2.13)

Smoking status		
​ Never	649/10 120	1.00 (referent)
​ Former	79/819	1.32 (1.02–1.71)
​ Current	181/1467	1.48 (1.22–1.79)

Alcohol drinking status		
​ Never	440/6637	1.00 (referent)
​ Former	104/800	1.55 (1.21–1.98)
​ Current	391/5197	1.04 (0.89–1.22)

Body-mass index		
​ <18.5 kg/m^2^	79/641	1.49 (1.14–1.93)
​ 18.5–24.9 kg/m^2^	636/8876	1.00 (referent)
​ ≥25.0 kg/m^2^	248/3423	0.98 (0.84–1.16)

Time spent walking per day		
​ <30 min	389/5036	0.93 (0.79–1.10)
​ 30 min–1 hr	277/4147	0.91 (0.76–1.09)
​ ≥1 hr	275/3623	1.00 (referent)

Lack of social support:		
​ (i) to consult when you are ​ in trouble	279/1349	2.20 (1.73–2.79)
​ (ii) to consult when you are ​ in bad physical condition	256/1254	1.38 (1.07–1.78)
​ (iii) to help with your daily ​ housework	310/2016	1.15 (0.93–1.42)
​ (iv) to take you to a hospital	218/1232	1.33 (1.05–1.67)
​ (v) to take care of you	305/2031	1.40 (1.13–1.74)

No participation in community activities		
​ (i) Activities of neighborhood ​ association	618/6833	1.26 (1.07–1.48)
​ (ii) Sports or exercise	702/7344	1.70 (1.43–2.02)
​ (iii) Volunteering	763/9303	0.97 (0.79–1.19)
​ (iv) Social gatherings	664/7327	1.20 (1.02–1.42)

Abbreviations: OR, odds ratio; CI, confidence interval.

^a^The K6 was used as an indicator of psychological distress,^3^^,^^4^ with a cut-off point of ≥13 out of 24 points.^15^

^b^In the multivariate models, all variables shown in Table 3 were adjusted for each other.

Among men aged 65 years or older, there was a loss of the significant associations with age, a history of diabetes mellitus, history of myocardial infarction, being a former smoker, being a current smoker, lacking social support for consultation when in bad physical condition, and lack of participation in community sports or exercise activities (Table 4). Among women aged 65 years or older, there was a loss of the significant associations with a history of diabetes mellitus, being a former smoker, being a current smoker, and lacking social support for help with daily housework (Table 5).

**Table 4.** Multivariate analysis of the association between psychological distress and demographic, medical, lifestyle, and social factors among men aged 65 years or older^a^

Variables	No. of persons with psychological distress /No. of participants	Multivariate OR (95% CI)^b^
Age group (years)		
​ 65–69	95/2323	1.00 (referent)
​ 70–74	114/2379	1.01 (0.75–1.35)
​ 75–79	105/1833	0.98 (0.72–1.33)
​ 80–84	65/925	1.01 (0.71–1.43)
​ ≥85	31/449	0.78 (0.49–1.22)

History of diseases		
​ Hypertension	194/3295	1.23 (0.99–1.53)
​ Diabetes mellitus	77/1128	1.25 (0.95–1.64)
​ Stroke	61/445	1.91 (1.39–2.62)
​ Myocardial infarction	44/544	1.33 (0.94–1.88)
​ Cancer	63/860	1.39 (1.03–1.87)

Smoking status		
​ Never	77/1862	1.00 (referent)
​ Former	222/3925	1.06 (0.80–1.40)
​ Current	90/1855	1.05 (0.76–1.47)

Alcohol drinking status		
​ Never	92/1646	1.00 (referent)
​ Former	149/1524	1.37 (1.03–1.83)
​ Current	154/4573	0.75 (0.57–1.00)

Body-mass index		
​ <18.5 kg/m^2^	35/343	1.56 (1.04–2.34)
​ 18.5–24.9 kg/m^2^	209/4597	1.00 (referent)
​ ≥25.0 kg/m^2^	91/1878	1.13 (0.86–1.47)

Time spent walking per day		
​ <30 min	234/2687	2.14 (1.58–2.88)
​ 30 min–1 hr	80/2767	0.95 (0.67–1.34)
​ ≥1 hr	63/2255	1.00 (referent)

Lack of social support:		
​ (i) to consult when you are ​ in trouble	112/1039	1.87 (1.35–2.58)
​ (ii) to consult when you are ​ in bad physical condition	68/614	0.90 (0.59–1.36)
​ (iii) to help with your daily ​ housework	100/1198	0.92 (0.66–1.28)
​ (iv) to take you to a hospital	70/572	1.77 (1.18–2.67)
​ (v) to take care of you	81/682	1.68 (1.16–2.43)

No participation in community activities		
​ (i) Activities of neighborhood ​ association	299/3693	1.82 (1.32–2.51)
​ (ii) Sports or exercise	285/3886	1.23 (0.92–1.64)
​ (iii) Volunteering	326/4641	1.64 (1.11–2.41)
​ (iv) Social gatherings	278/3477	1.35 (1.00–1.82)

Abbreviations: OR, odds ratio; CI, confidence interval.

^a^The K6 was used as an indicator of psychological distress,^3^^,^^4^ with a cut-off point of ≥13 out of 24 points.^15^

^b^In the multivariate models, all variables shown in Table 4 were adjusted for each other.

**Table 5.** Multivariate analysis of the association between psychological distress and demographic, medical, lifestyle, and social factors among women aged 65 years or older^a^

Variables	No. of persons with psychological distress /No. of participants	Multivariate OR (95% CI)^b^
Age group (years)		
​ 65–69	145/2768	1.00 (referent)
​ 70–74	182/2863	1.06 (0.84–1.34)
​ 75–79	176/2334	1.08 (0.84–1.37)
​ 80–84	149/1422	1.31 (1.01–1.69)
​ ≥85	149/1072	1.49 (1.14–1.96)

History of diseases		
​ Hypertension	387/4609	1.14 (0.98–1.33)
​ Diabetes mellitus	95/1094	1.01 (0.80–1.28)
​ Stroke	62/336	1.86 (1.37–2.51)
​ Myocardial infarction	52/382	1.46 (1.06–2.00)
​ Cancer	84/701	1.61 (1.25–2.08)

Smoking status		
​ Never	620/8138	1.00 (referent)
​ Former	34/346	0.94 (0.64–1.39)
​ Current	25/290	0.92 (0.59–1.43)

Alcohol drinking status		
​ Never	548/7136	1.00 (referent)
​ Former	65/534	1.42 (1.06–1.91)
​ Current	80/1324	1.01 (0.78–1.31)

Body-mass index		
​ <18.5 kg/m^2^	73/553	1.38 (1.04–1.83)
​ 18.5–24.9 kg/m^2^	387/5388	1.00 (referent)
​ ≥25.0 kg/m^2^	178/2795	0.84 (0.70–1.02)

Time spent walking per day		
​ <30 min	473/4335	1.73 (1.37–2.18)
​ 30 min–1 hr	176/3469	1.05 (0.81–1.35)
​ ≥1 hr	103/2299	1.00 (referent)

Lack of social support:		
​ (i) to consult when you are ​ in trouble	143/697	1.75 (1.29–2.37)
​ (ii) to consult when you are ​ in bad physical condition	116/522	1.63 (1.14–2.31)
​ (iii) to help with your daily ​ housework	168/1282	1.13 (0.86–1.48)
​ (iv) to take you to a hospital	106/690	1.20 (0.88–1.63)
​ (v) to take care of you	207/1589	1.50 (1.18–1.90)

No participation in community activities		
​ (i) Activities of neighborhood ​ association	610/6034	1.38 (1.09–1.75)
​ (ii) Sports or exercise	623/5950	2.22 (1.72–2.85)
​ (iii) Volunteering	673/7419	1.69 (1.18–2.43)
​ (iv) Social gatherings	598/5800	1.57 (1.24–1.99)

Abbreviations: OR, odds ratio; CI, confidence interval.

^a^The K6 was used as an indicator of psychological distress,^3^^,^^4^ with a cut-off point of ≥13 out of 24 points.^15^

^b^In the multivariate models, all variables shown in Table 5 were adjusted for each other.

When we further added current employment status and the duration of education as covariates in the multivariate models, as shown in Table 2 and Table 3, the multivariate-adjusted OR (95% CI) for psychological distress associated with being currently employed was 1.65 (1.30 to 2.09) among men and 1.10 (0.84 to 1.28) among women, respectively, and 0.82 (0.68 to 0.98) among men and 0.93 (0.80 to 1.09) among women for longer duration of education.

In addition, we analyzed the data using different cut-off points (≥9/24, ≥11/24, and ≥15/24), but the results did not substantially change in an analysis of all participants or in stratified analyses (data not shown).

## DISCUSSION

The use of general population surveys to measure the extent of mental illness presents many challenges because the diagnostic tools employed tend to be lengthy and cumbersome.^17^^,^^18^ The results of the present study suggest that use of the K6 scale as a proxy indicator of mental health impairments contributes to the investigation of factors associated with mental health at the population level.

On the basis of baseline cross-sectional data from a new, large, population-based, prospective cohort study, we found that female sex, young and old age, history of hypertension, history of diabetes mellitus, history of stroke, history of myocardial infarction, history of cancer, current smoking, former alcohol drinking, low BMI, shorter daily walking time, lack of social support, and lack of participation in community activities were all associated with psychological distress, even in multivariate analysis. Nevertheless, stratified analysis by sex and age categories (40 to 64 years, 65 years or older) revealed some differences among strata. The present findings indicate that factors associated with psychological distress differ between men and women, and also between middle-aged and elderly people.

We found that, as compared to men, women were more likely to have psychological distress, even in multivariate analysis, which was consistent with 2 previous US studies that used the K6.^5^^,^^8^ Several studies have also shown that women have a higher risk of anxiety and mood disorders, suggesting that many factors, such as female hormones, personality, coping skills, and sociocultural roles, play a direct role in anxiety and mood disorders, as do socioeconomic status and comorbid conditions.^19^^–^^21^

The association of advanced age with psychological distress was substantially attenuated in multivariate analysis, suggesting that the high OR in the univariate model might be due to other variables shown in Table 1. Nevertheless, there was still a U-shaped association between age category (5-year categories from 40 to 44 years to ≥85 years) and the prevalence of psychological distress, with a nadir for those aged 65 to 69 years. This pattern of association is consistent with that of a previous study.^5^ In contrast, stratified analysis revealed no apparent association between age and psychological distress among men aged 65 years or older, which suggests that age alone was not associated with psychological distress among men in this age category.

The associations of psychological distress with a history of serious disease were as unsurprising. Similar associations were also reported in a survey conducted in the United States.^5^ The strong association between a history of stroke and psychological distress may be due to post-stroke depression.^22^ Nevertheless, stratified analyses revealed some differences among sex and age categories. A history of hypertension was not significantly associated with psychological distress in any stratum. Although not significant, point estimates for history of hypertension were all above unity, which is suggestive of relatively small differences among strata. A history of diabetes mellitus was significantly associated with psychological distress only among men aged 40 to 64 years, indicating the potential burden of this disease among middle-aged men. A history of stroke was not significantly associated with psychological distress among women aged 40 to 64 years, but the point estimate was similar to that among women aged 65 years or older, suggesting that the disease burden was similar for women in these 2 age groups. The significant association between a history of myocardial infarction and psychological distress disappeared among men aged 40 to 64 years and 65 years or older, suggesting a potential sex difference in disease burden. A history of cancer was not significantly associated with psychological distress among men aged 40 to 64 years, although the reason for this was unclear.

We also found that former smoking, current smoking, former alcohol drinking, being underweight, and shorter daily walking time were associated with a higher prevalence of psychological distress. In contrast, we observed a lower prevalence among participants with a moderate daily walking time. The results for former smoking,^8^ current smoking,^5^^,^^8^ and being underweight^5^ were consistent with previous studies. Stratified analyses yielded reduced point estimates for current smoking among men aged 65 years or older and women aged 65 years or older, suggesting that the smoking habit itself, as well as related factors, was not strongly associated with psychological distress among persons aged 65 years or older, in contrast to those aged 40 to 64 years.

Among the variables studied, lack of social support was most strongly associated with a high prevalence of psychological distress, even in multivariate analysis. Although this is the first large population-based epidemiological study using the K6 in an Asian country, previous studies^23^^,^^24^ have used other mental health scales, such as the Geriatric Depression Scale (GDS)^25^ among Japanese populations. Koizumi et al reported that negative responses to the questions “Do you have someone with whom you can consult when you are in trouble?” and “Do you have someone who can take care of you when you are ill in bed?” were significantly associated with an increase in the risk of depression.^23^^,^^24^ The finding is consistent with, and supports, the present results for persons aged 65 years or older. The depressive symptoms detected by the GDS and the psychological distress detected by the K6 reflect common underlying factors.

Although lack of social support was strongly associated with a high prevalence of psychological distress, the significant association that had been found with 3 components of deficient social support disappeared on multivariate analysis among men aged 40 to 64 years (Table 2). However, lack of social support for consultation when in trouble remained strongly associated with psychological distress. These results appear to underline the importance of such support among men aged 40 to 64 years.

The association of lack of participation in community activities with psychological distress was substantially attenuated in multivariate analysis, indicating that the high OR in the univariate models could be largely explained by other variables shown in Table 1. Nevertheless, the significant increases in OR in the multivariate model indicate that lack of participation in community activities may also be associated with mental health. Stratified analysis revealed that the significant association between lack of participation in community activities in a neighborhood association disappeared among men aged 40 to 64 years, indicating the relatively low influence of neighborhood community on middle-aged men. Also, the significant association with lack of participation in volunteer activities disappeared among men and women aged 40 to 64 years, but the point estimate among men was similar to that among the total population. However, the lower point estimate on multivariate analysis suggests a relatively weak association with participation in volunteer activities among women aged 40 to 64 years.

Our data showed that being currently employed was associated with a high odds of psychological distress, and that a longer duration of education was associated with a lower odds of psychological distress, among men aged 40 to 64 years. Although the reason is unclear, our data suggest that some socioeconomic factors, such as employment and education, are important among men aged 40 to 64 years.

Our study did have some limitations. First, because of the cross-sectional design, the direction of causation for the associations observed in this report cannot be inferred from the data. Prospective studies that measure the K6 in respondents at baseline, follow the respondents over time, and measure the K6 at the end of follow-up, are needed to clarify these causal relationships.

Second, because the response rate was not high (64.5%), the respondents may not be a representative sample of the source population of Ohsaki City residents. The response rates among men and women aged 40 to 64 years were lower (54.9% and 60.4%, respectively) than those among men and women aged 65 years or older (77.1% and 73.2%, respectively). These relatively low response rates, especially among participants aged 40 to 64 years, should be kept in mind when interpreting the results from prospective, as well as cross-sectional, analyses.

Third, because the K6 does not provide information about the specific psychiatric conditions of respondents, it is difficult to identify what is being measured. However, the particular symptoms included in the K6 make it likely that severe, disabling, mood and anxiety disorders are being identified.^3^^,^^4^^,^^15^^,^^16^ Although the K6 focuses on nonspecific psychological distress, the majority of cases detected by this instrument would meet the criteria for certain mental health disorders specified in the Diagnostic and Statistical Manual of Mental Disorders, Fourth Edition.^3^^,^^15^

Finally, no scales, including the present one, have been adequately validated for use as social support questionnaires in the Japanese population. Also, the first question in the questionnaire, “Do you have someone with whom you can consult when you are in trouble?”, might be construed to include the participant’s family, which may not qualify as social support.

In conclusion, the findings of this cross-sectional study demonstrate that the factors associated with psychological distress differ between men and women, and also between middle-aged and elderly people. These findings underline the importance of considering sex and age categories when attempting to minimize psychological distress in community-dwelling populations. To our knowledge, this is the first large population-based epidemiological study to use the K6 in an Asian country.
